# Multilocus Sequence Typing Reveals a New Cluster of Closely Related *Candida tropicalis* Genotypes in Italian Patients With Neurological Disorders

**DOI:** 10.3389/fmicb.2018.00679

**Published:** 2018-04-06

**Authors:** Fabio Scordino, Letterio Giuffrè, Giuseppina Barberi, Francesca Marino Merlo, Maria Grazia Orlando, Domenico Giosa, Orazio Romeo

**Affiliations:** ^1^Scientific Institute for Research, Hospitalization and Health Care (IRCCS), Centro Neurolesi “Bonino-Pulejo”, Messina, Italy; ^2^Department of Veterinary Sciences, Division of Animal Production, University of Messina, Messina, Italy; ^3^Department of Chemical, Biological, Pharmaceutical and Environmental Sciences, University of Messina, Messina, Italy

**Keywords:** *Candida tropicalis*, candidemia, multilocus sequence typing (MLST), genetic diversity, population structure, neurological patients

## Abstract

*Candida tropicalis* is a pathogenic yeast that has emerged as an important cause of candidemia especially in elderly patients with hematological malignancies. Infections caused by this species are mainly reported from Latin America and Asian-Pacific countries although recent epidemiological data revealed that *C. tropicalis* accounts for 6–16.4% of the *Candida* bloodstream infections (BSIs) in Italy by representing a relevant issue especially for patients receiving long-term hospital care. The aim of this study was to describe the genetic diversity of *C. tropicalis* isolates contaminating the hands of healthcare workers (HCWs) and hospital environments and/or associated with BSIs occurring in patients with different neurological disorders and without hematological disease. A total of 28 *C. tropicalis* isolates were genotyped using multilocus sequence typing analysis of six housekeeping (*ICL1, MDR1, SAPT2, SAPT4, XYR1*, and *ZWF1*) genes and data revealed the presence of only eight diploid sequence types (DSTs) of which 6 (75%) were completely new. Four eBURST clonal complexes (CC2, CC10, CC11, and CC33) contained all DSTs found in this study and the CC33 resulted in an exclusive, well-defined, clonal cluster from Italy. In conclusion, *C. tropicalis* could represent an important cause of BSIs in long-term hospitalized patients with no underlying hematological disease. The findings of this study also suggest a potential horizontal transmission of a specific *C. tropicalis* clone through hands of HCWs and expand our understanding of the molecular epidemiology of this pathogen whose population structure is still far from being fully elucidated as its complexity increases as different categories of patients and geographic areas are examined.

## Introduction

Globally, each year, 300 million people of all ages suffer from a serious fungal infection and over 1.5 million of these people die as a result of such diseases (Brown et al., 2012; Del Poeta, 2016). According to the latest World Health Organization’s fact sheets^1^, this mortality rate currently exceeds that of other serious human infectious diseases, such as malaria (445,000 deaths in 2016), hepatitis B (887,000 deaths in 2015), or HIV/AIDS (1 million deaths in 2016), and is rather similar to that of tuberculosis (1.7 million deaths in 2016).

The impact of invasive fungal infections on healthcare systems has also heavy costs, both in terms of human lives and financial resources (Drgona et al., 2014). In this context, *Candida* species hold a predominant position by representing the sixth most common cause of all bloodstream infections (BSIs) acquired in the intensive care units (ICUs) of European hospitals (European Centre for Disease Prevention and Control [ECDC], 2016).

Globally, invasive candidiasis affects more than 700,000 people worldwide every year and, with an unacceptably high mortality rate over 40%, is the cause of more than 280,000 deaths (Bongomin et al., 2017). Moreover, the annual incidence rates of candidemia have been reported to be between 2 and 14 cases per 100,000 persons in several population-based epidemiological studies (Quindós, 2014; Kullberg and Arendrup, 2015) but a recent survey has revealed a substantial increase of this disease in Italy (21.8 cases per 100,000 persons) (Bassetti et al., 2017) by showing an incidence higher than that of Pakistan (21 cases per 100,000), previously considered as one of the countries with the highest prevalence of candidemia (Bongomin et al., 2017).

In Italy, *Candida* species account for almost 10% of all ICU-acquired BSI (European Centre for Disease Prevention and Control [ECDC], 2016) with *Candida albicans* being the most frequently encountered species followed by *Candida parapsilosis, Candida glabrata, Candida krusei*, and *Candida tropicalis* (European Centre for Disease Prevention and Control [ECDC], 2013). However, several Italian studies have shown significant regional differences in the prevalence of pathogenic *Candida* spp., and often non-albicans *Candida* (NAC) species cause ∼50% of the *Candida* BSIs (Bassetti et al., 2011; Prigitano et al., 2016) or even outrank *C. albicans* in some hospitals, especially in those of the southern regions (Montagna et al., 2010, 2013; Delfino et al., 2014; Caggiano et al., 2015).

Among the different NAC species, *C. tropicalis* is of particular importance as it emerged as a major cause of nosocomial candidemia in the elderly worldwide, particularly in the Asia-Pacific region and Latin America (Kothavade et al., 2010; Negri et al., 2012; Quindós, 2014; Motoa et al., 2017; Pu et al., 2017). This fungus is, generally, less often encountered in the rest of the world (Quindós, 2014) but recent surveillance studies in Italy revealed that this species accounts for 6–16.4% of the candidemia cases (Bassetti et al., 2013; Montagna et al., 2013; Caggiano et al., 2015; Tedeschi et al., 2016; De Francesco et al., 2017) by representing a relevant issue given the high mortality associated with its infections (Muñoz et al., 2011; Negri et al., 2012; Caggiano et al., 2015).

*Candida tropicalis* is a diploid yeast that together with *C. albicans, C. parapsilosis*, and other less relevant *Candida* pathogens forms part of the so-called *Candida* CTG clade in which the CUG codon is translated as serine rather than leucine (Papon et al., 2013). Genetically, the population structure of this fungus appears to be quite heterogeneous, and more complex (Wu et al., 2012; Al-Obaid et al., 2017; Wu et al., 2017) than the one previously described by Tavanti et al. (2005) using multilocus sequence typing (MLST) technique. However, compared to the *C. albicans* MLST database^2^, which contains genetic data from over 4000 isolates, the number of *C. tropicalis* isolates genotyped so far is still too small to provide a good estimate of the pattern of the genetic variation occurring in this species. In fact, on 18 December 2017, the *C. tropicalis* MLST database^3^ contained 500 sequences, arranged in 717 diploid sequence types (DSTs), obtained from 880 worldwide isolates. Of these, only 16% (143/880 isolates) came from Europe (124 United Kingdom; 10 Belgium; 3 Germany; 3 Netherlands; and 1 each from Greece, Spain, and Sweden, respectively) and no Italian *C. tropicalis* isolates have never been submitted in the public database. This greatly limits our understanding of the genetic diversity of *C. tropicalis* from different geographical areas and, consequently, the global molecular epidemiology of this important human fungal pathogen is, at present, largely unknown.

In this study we report, for the first time, MLST-genotyping data of a panel of Italian *C. tropicalis* isolates recovered from clinical samples and hospital environments and describe the existence of a specific cluster of new closely related MLST genotypes transmitted to hospitalized patients probably via contaminated healthcare workers’ (HCWs) hands.

## Materials and Methods

### Fungal Isolates and Phenotypic Identification

A total of 28 *C. tropicalis* isolates were examined in this study (**Table 1**). These isolates were recovered during an ongoing surveillance project funded by the Italian Ministry of Health for the prevention and control of healthcare-associated fungal infections. According to this project, all *C. tropicalis* recovered from blood samples or catheters tips by diagnostic microbiology laboratory of the hospital were subjected to molecular identification and genotyping.

**Table 1:** *Candida tropicalis* isolates and multilocus sequence typing profiles generated using six loci of the MLST scheme.

PT	W	Isolate n°	Sample	Isolation date	MLST loci						DST
					ICL1	MDR1	SAPT2	SAPT4	XYR1	ZWF1	
P1	C	IRCCS-1	Blood	21/11/2015	17	93	29	83	138	30	747
P1	C	IRCCS-2	PICC	21/11/2015	17	93	29	83	138	30	747
P2	C	IRCCS-3	Blood	21/11/2015	1	44	12	7	94	22	333
P3	B	IRCCS-7	Blood	02/12/2015	17	93	29	82	138	30	748
P3	B	IRCCS-8	PICC	02/12/2015	17	93	29	82	138	30	748
P4	A	IRCCS-14	Blood	04/01/2016	1	17	2	14	53	3	750
P5	C	IRCCS-15	Blood	05/01/2016	17	93	29	83	138	30	747
P6	C	IRCCS-23	Blood	22/06/2016	17	93	29	83	138	30	747
P6	C	IRCCS-24	PICC	22/06/2016	17	93	29	83	138	30	747
P7	B	IRCCS-28	Blood	16/07/2016	1	7	3	10	9	3	751
P7	B	IRCCS-29	PICC	16/07/2016	1	7	3	10	9	3	751
P8	C	IRCCS-31	Blood	28/07/2016	17	93	29	83	138	30	747
P8	C	IRCCS-32	PICC	28/07/2016	17	93	29	83	138	30	747
P9	C	IRCCS-37	Blood	15/03/2017	17	93	29	83	138	30	747
P9	C	IRCCS-38	PICC	15/03/2017	17	93	53	83	138	30	749
P9	C	IRCCS-39	PICC	24/03/2017	17	93	29	83	138	30	747
P10	C	IRCCS-40	Blood	18/03/2017	1	44	12	7	94	22	333
P10	C	IRCCS-42	Blood	27/03/2017	1	44	12	7	94	22	333
P10	C	IRCCS-43	Blood	06/04/2017	1	44	12	7	94	22	333
P11	C	IRCCS-46	Blood	11/04/2017	1	17	2	14	100	3	359
P11	C	IRCCS-47	PICC	11/04/2017	1	17	2	14	100	3	359
P12	C	IRCCS-51	Blood	17/04/2017	17	93	29	83	2	30	759
P12	C	IRCCS-52	PICC	17/04/2017	17	93	29	83	2	30	759
–	B	B4BR3	DH	01/03/2016	1	44	12	7	94	22	333
–	C	C1T1V	BT	16/12/2015	17	93	29	83	138	30	747
–	A	ALOG1	HCW	19/04/2016	1	44	12	7	94	22	333
–	C	OSS1C-R	HCW	19/10/2016	17	93	29	83	138	30	747
–	C	IRXLP2	BH	16/12/2015	17	93	29	83	138	30	747

*PT, patient; W, ward; DST, diploid sequence type assigned by *C. tropicalis* MLST database; PICC, peripherally inserted central catheter; DH, door handle; BT, bedside table; HCW, healthcare worker; BH, bed handles.*

Twenty-three *C. tropicalis* isolates were obtained from blood samples and peripherally inserted central catheters (PICCs) of 12 patients hospitalized in three different wards of the IRCCS Centro Neurolesi Bonino-Pulejo, Messina, Italy, during a 17-month period (November 2015 to April 2017) (**Table 1**). Five additional isolates were collected during the microbiological surveillance activities undertaken in the hospital from ward environments and hands of two HCWs (social-health operator and logopedist) (**Table 1**). The study was approved by the ethical committee of the IRCCS Centro Neurolesi Bonino-Pulejo (study code: HCW_GR2011-02347606; protocol number: E12/17) and all experiments were performed in accordance with relevant guidelines and regulations.

All *C. tropicalis* isolates were initially, phenotypically, identified using CHROMagar *Candida* medium (Becton Dickinson, Italy) and Vitek 2 yeast identification system (bioMérieux, Italy) following manufacturer’s recommendations.

The yeast isolates were sub-cultured and stored on Sabouraud dextrose agar until molecular testing.

### Antifungal Drug Susceptibility Testing

The susceptibility of *C*. *tropicalis* isolates to six antifungal drugs (fluconazole, voriconazole, amphotericin B, 5-flucytosine, micafungin, and caspofungin) was determined by using the Vitek 2 system according to the manufacturer’s instructions (bioMérieux, Italy). The results were interpreted according to the revised interpretive susceptibility breakpoints as recommended by clinical laboratory standards institute (CLSI) document M27-S4 (Clinical and Laboratory Standards Institute [CLSI], 2012).

### DNA Extraction and Molecular Identification of *C. tropicalis* Isolates

The identity of the yeast isolates was confirmed by sequencing the ITS1-5,8S-ITS2 region of the ribosomal DNA (rDNA) (Leaw et al., 2006). Genomic DNA was extracted from yeast cells using mechanical glass-beads disruption method followed by traditional phenol/chloroform extraction and ethanol precipitation as described in Müller et al. (1998).

PCR amplifications were carried out using the DreamTaq^TM^ PCR master mix (Fermentas, Milan, Italy) to which were only added 0.5 μg of genomic DNA template and 0.5 μM of each primer: ITS1-TCCGTAGGTGAACCTGCGG and ITS4-TCCTCCGCTTATTGATATGC (White et al., 1990). The PCR cycling parameters used consisted of an initial heating to 95°C for 5 min, 35 cycles of denaturation at 94°C for 1 min, annealing at 53°C for 40 s, and extension at 72°C for 45 s, followed by a final extension step of 10 min at 72°C. Following PCR, each product was purified with the QIAquick PCR Purification Kit (Qiagen, Italy) and sequenced at Eurofins Genomics, Ebersberg, Germany^4^ using the same primers used for PCR.

The identity of the nucleotide sequences was assessed by BLASTN searches^5^ in the Genbank database using a sequence identity value of ≥99% for species-level identification.

Two representative rDNA sequences have been deposited in the Genbank database under the following accession numbers: MH000022 and MH000023.

### Multilocus Sequence Typing (MLST) of Italian *C. tropicalis* Isolates

The MLST scheme used for *C. tropicalis* genotyping was based on sequence analysis of six housekeeping genes: *ICL1, MDR1, SAPT2, SAPT4, XYR1*, and *ZWF1* (Tavanti et al., 2005). For each isolate, six separate PCR amplifications were performed using the DreamTaq^TM^ PCR master mix, 100 ng of genomic DNA template, and 10 μM forward and reverse primers (Tavanti et al., 2005). Primers and conditions used for PCR amplifications were the same as those previously described by Tavanti et al. (2005). The amplified products were purified and bidirectionally sequenced as described above for ITS sequencing.

Sequencing chromatograms were visually reviewed using the FinchTV v1.4 software to detect call errors and to determine the positions of heterozygous polymorphisms. Single-nucleotide polymorphisms were confirmed by examination of both forward and reverse sequence traces. The one-letter IUPAC nucleotide code was used in sequence analysis. The sequence data at each locus were compared with sequences deposited into the public *C. tropicalis* MLST database^6^ for assigning the allele numbers.

For each single isolate, the DST was defined by the composite profile of all six allele numbers. New allele sequences were deposited into the *C. tropicalis* MLST database and alleles numbers were provided by the curator of the database: Prof. Frank C. Odds.

### Phylogenetic and Population Structure Analysis

Phylogenetic analysis by unweighted pair-group method with arithmetic average (UPGMA), using the *p*-distance model, was conducted with MEGA version 7 software (Kumar et al., 2016) according to Tavanti et al. (2005). Before UPGMA clustering, nucleotide sequences from the six sequenced loci were concatenated and modified as described by Tavanti et al. (2005) to label homozygous and heterozygous polymorphic sites in order to allow the analysis of diploid sequence data with MEGA7. The significance of the UPGMA cluster nodes was determined by bootstrapping with 1000 pseudo-replicates.

A total of 635 validated DSTs (for which at least one isolate was published in the *C. tropicalis* MLST database) were downloaded and included in the phylogenetic analysis.

A newick file of the resulting UPGMA tree, along with geospatial and genetic information for each isolate, was also loaded into a MicroReact project (Argimón et al., 2016) for displaying the phylogeography and the current molecular epidemiology of *C. tropicalis* on a global scale.

To determine the MLST clonal complexes (CCs) where Italian isolates belonged, the e-BURST analysis was performed using the goeBURST algorithm implemented in the PHYLOViZ 2.0 software (Nascimento et al., 2017). Isolates were considered as belonging to the same CC if sharing five out of six genes [single-locus variant (SLV) analysis] used for MLST.

### Data Availability

All data generated during this study are included in this published article and are also publicly available and accessible online at *C. tropicalis* MLST database^6^ and at Microreact interactive viewer^7^.

## Results

### Characteristics of the Patients

In this study, most of the patients (9/12; 75%) with *C. tropicalis* candidemia were from ward C. Two patients were hospitalized in the ward B and only 1 was from the ward A (**Table 1**).

The 12 patients showed different underlying diseases or acquired brain injuries, from stroke (P1, P9, and P10) to post-anoxic encephalopathy (P2), intracranial injury (P3, P4, and P11), post-traumatic cerebral hemorrhage (P5, P6, and P8), intracranial epidermoid cysts (P7), and meningioma (P12).

The age of the patients ranged from 45 to 75 years (mean 62.5 ±*SD* 10.26) and half of them were >65 years old. The ratio of male to female was nearly equal (five males and seven females).

All the patients had urinary catheters and eight of them also had a peripherally inserted central catheter placed (**Table 1**).

### Phenotypic and Molecular Identification and Antifungal Drug Susceptibility Testing of *C. tropicalis* Isolates

All *C. tropicalis* recovered in this study grew as blue colonies on CHROMagar *Candida* and their identity was confirmed using both Vitek 2 system and ITS sequencing. BLAST analysis of the whole ITS1-5,8S-ITS2 sequence showed <1% nucleotide difference with the corresponding sequences from the reference strains: *C*. *tropicalis* ATCC 750 (Genbank: KU729064) and ATCC 13803 (Genbank: KU729076).

Multiple yeast isolates were recovered from the same patient and collected on the same day or few days apart (patients P9 and P10) (**Table 1**). However, most of the patients yielding multiple *C. tropicalis* isolates (8/9; ∼89%) showed positive cultures from both blood and catheters tips (**Table 1**). Only one patient (P10) showed more than one positive blood culture. The remaining isolates were individually collected from different patients (P2, P4, and P5), HCWs, and ward environments (door handle, bedside table, and bed handles) (**Table 1**).

All *C. tropicalis* isolates were sensitive to the six antifungal drugs tested. We only observed a slight variation between minimum inhibitory concentration (MIC) values for amphotericin B which ranged from 0.25 (four isolates: IRCSS 28, 29, 46, and 47) (**Table 1**) to 0.5 μg/ml (for the remaining 24 isolates) whereas for the other antifungal agents tested, the MIC values (fluconazole: <1 μg/ml; voriconazole: <0.12 μg/ml; 5-flucytosine: <1 μg/ml; micafungin: <0.06 μg/ml; caspofungin: <0.25 μg/ml) showed no variation among the isolates.

### Genetic Diversity and Population Structure of Italian *C. tropicalis* Isolates by MLST

Sequencing of 370–525 bp fragments from the coding region of all the six genes included in the MLST scheme resulted in a total of 2677 aligned nucleotides for each *C. tropicalis* isolate. A total of 25 different alleles were found among the six sequenced DNA fragments (**Table 1**) and *XIR1* locus was the most informative with six diverse alleles found followed by *SAPT2* and *SAPT4* loci (five alleles each), *MDR1* (four alleles), *ZWF1a* (three alleles), and *ILC1* (two alleles). Overall 137 polymorphic sites were detected among all sequenced loci (*ICL1*: 13; *MDR1*: 18; *SAPT2*: 38; *SAPT*4: 38; *XIR*1: 20; *ZWF1*a: 10). Four alleles (allele 53 for *SAPT2* locus, alleles 82 and 83 for *SAPT4* locus, and allele 138 for *XIR1* locus) (**Table 1**) were completely new and were submitted in the *C*. *tropicalis* MLST database for assigning new allele/genotype numbers (**Table 1**).

When all six alleles numbers were combined, a total of eight different DSTs were obtained from the 28 *C. tropicalis* isolates (**Table 1**). Interestingly, six (DSTs: 747-51 and 759) of these eight genotypes (75%) were novel to the MLST central database and one genotype (DST747) was the most frequently observed (12/28 isolates; ∼42.9%) followed by the genotype DST333 (6/28 isolates; ∼21.4%).

Four genotypes (DSTs 748, 751, 359, and 759) were found with an individual incidence of ∼7.1% (two isolates each) and each of them was recovered from a single patient (**Table 1**). The remaining two genotypes (new DSTs 749 and 750; incidence 3.6% each) were found to be individual genotypes (singletons) recovered only once in single patients (P4 and P9; **Table 1**). Interestingly, the DST749 from patient P9 was recovered from a PICC tip while the blood sample collected on the same day showed the presence of the DST747 genotype suggesting a possible mixed infection. This was supported by the fact that the culture of a new PICC tip obtained after 9 days yielded the DST747 type (**Table 1**).

However, if we consider that the identical DSTs collected from the same patient on the same day (P1, P3, P6, P7, P8, P11, and P12) could represent a single epidemic clone, rather than a coincidental emergence of unrelated strains, the number of the total isolates drops to 21 causing only a slight variation of the previously reported genotype frequencies (DST747: 9/21; 42.8%, DST333: 6/21; 28.5%, DSTs 748–751, 359, and 759: 1/21; ∼4.8% each).

In addition, we noticed a differential distribution of the MLST genotypes between the three hospital wards considered. The most common DST747 was restricted to clinical and environmental samples from the ward C whereas DSTs 748 and 751 were only recovered from blood samples and PICCs tips of two different patients (P3 and P7) hospitalized in the ward B. The DST750 was exclusively found in a single patient (P4) of the ward A (**Table 1**). There were no isolations of DSTs 748, 750, and 751 from hospital environments or HCWs working in the wards A and B which, on the contrary, resulted colonized by a distinct MLST genotype (DST333) that was never recovered from clinical samples from these units (**Table 1**).

**FIGURE 1:**
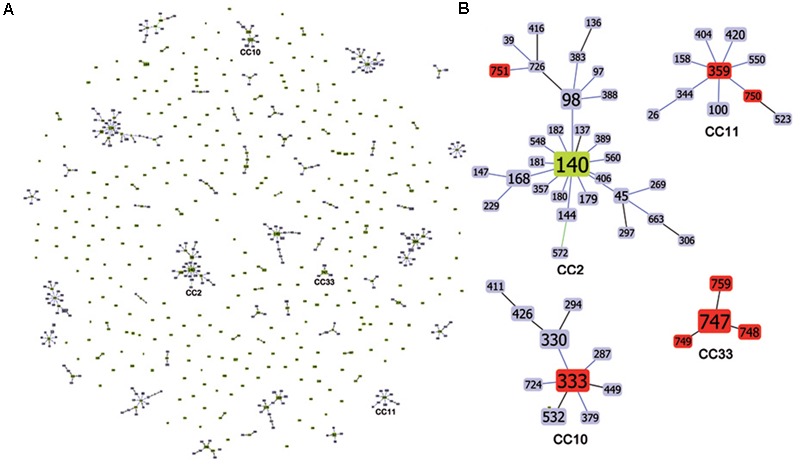
Genetic population structure of *C. tropicalis*. **(A)** Population snapshot obtained by goeBURST analysis using the 635 confirmed *C. tropicalis* DSTs currently deposited in the MLST database; DSTs are linked when they differ in just one of the six loci used for MLST (SLV analysis). The lengths of lines are not significant. Single DSTs represent singletons. **(B)** goeBURST clonal clusters CC2, CC10, CC11, and CC33 containing all the DSTs (in red) found in this study. For the CC2 the putative founding genotype is indicated in green. The size of each DST reflects the number of isolates with that DST in the input data set used for the analysis.

**FIGURE 2:**
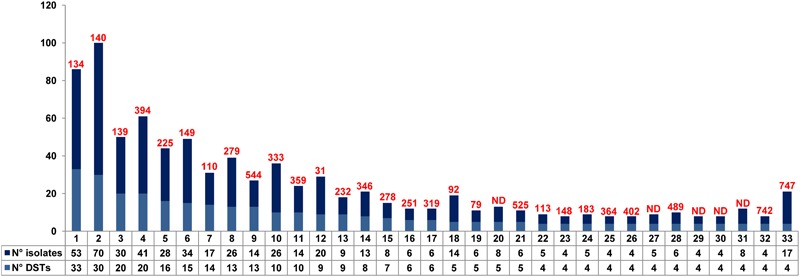
Details of the eBURST clonal clusters containing four or more DSTs. For each of the 33 CCs, the total number of DSTs and isolates is reported. The putative founding DST is also illustrated (in red) above the bars. ND = no DST could be determined as the putative founding genotype.

The MLST allelic profile data were further analyzed by using the goeBURST algorithm for inferring genetic relationships between the 28 Italian isolates and 880 isolates deposited in the *C*. *tropicalis* MLST database (as of 18 December 2017). The dataset contained a total of 635 confirmed DSTs that were grouped into 71 CCs by their similarity to a central allelic profile (ancestral or founding genotype) and 243 singletons. Data obtained by goeBURST analysis are depicted in **Figure 1**. Twenty-two clusters (CCs 1–22) contained 50% of the isolates included in the database (**Figure 2**). Details of the clonal clusters containing at least four DSTs are summarized in **Figure 2**.

All clonal clusters (CC2, CC10, CC11, and CC33) containing the DSTs found in this study are illustrated in **Figure 1**. Of the 8 DSTs representing the 28 Italian *C. tropicalis* isolates, 4 DSTs (333, 359, 750, and 751) were grouped in three different clusters (CC2, CC10, and CC11) (**Figure 1**).

The large cluster CC2 contained a total of 30 DSTs including one (DST751) from this study (**Figure 1**) whereas the CC10 had a total of 10 DSTs with the DST333 (the second most common genotype found here) as the putative founding type (**Figure 1**). All DSTs included in this latter cluster were formerly reported only from Asia while among those characterizing the CC2, the DST39 was found exclusively in the United States, the DST97 in Europe (United Kingdom), and the DSTs 45 and 98 in Europe/Asia and Latin America/Asia, respectively.

The CC11 contained 10 DSTs including 2 DSTs (359 and 750) from this study. The DST359 was predicted to be the founder of this CC (**Figure 1**). Except for the European DSTs 26 (Netherlands) and 750 (Italy) and the DST100 recovered only in Latin America (Colombia), all other MLST genotypes were found in the Asian Continent.

The remaining four genotypes (new DSTs 747, 748, 749, and 759) composed a single well-defined Italian clonal cluster (CC33) with the most commonly recovered type, the DST747, as putative founding genotype (**Figure 1** and **Table 1**).

**FIGURE 3:**
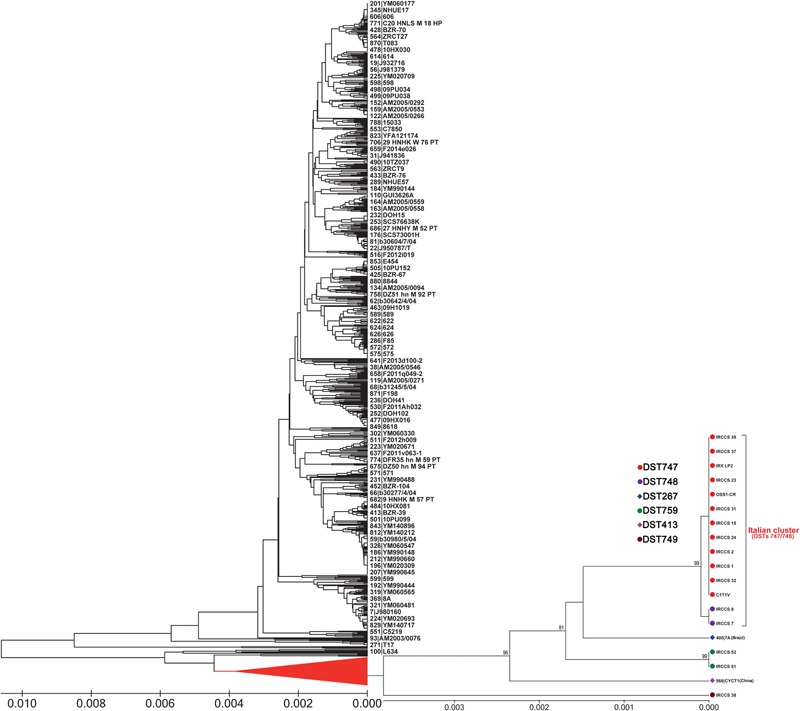
UPGMA dendrogram showing the genetic relationships of the Italian *C. tropicalis* isolates with 880 isolates available from the central MLST database (as of October 23, 2017). The evolutionary distances were computed using the *p*-distance method and are in the units of the number of base differences per site. For clarity, the subtree showing the UPGMA Italian cluster and all DSTs included in the goeBURST complex CC33 is illustrated on the right. Bootstrap support values above 80% are indicated at the nodes.

The existence of a specific Italian lineage was also confirmed by UPGMA analysis which grouped all DST747 and DST748 isolates in a single well-supported clade (bootstrap: 99%) (**Figure 3**), closely related to a Brazilian isolate (7A; DST267) that was not included in the CC33 generated by goeBURST analysis (**Figures 1, 3**). Conversely, other Italian CC33 isolates with DSTs 749 and 759, including a non-CC33 Chinese isolate (CYCT1; DST413) from feces (**Figure 1**), were placed at sufficient phylogenetic distance to be outside the UPGMA cluster formed by both DSTs 747 and 748 isolates (**Figure 3**). These latter two DSTs differ only at *SAPT4* locus which shows two different alleles (allele 83 in DST747 and 82 in DST748) (**Table 1**) that share 99.74% of nucleotide identity (only one difference at nucleotide 267: Y→C) while the genetic differences observed between the genotype 747 (or 748) and the DSTs 749 and 759 concern *SAPT2* and *XYR1* loci, respectively (**Table 1**). There were nucleotide identities of 95.41%, between the *XYR1* allele 2 (DST759) and 138 (DSTs 747 or 748), and 92.95% between the *SAPT2* allele 53 (DST749) and 29 (DSTs 747 or 748).

A fully interactive version of our UPGMA phylogenetic tree, including geospatial information and other metadata regarding all *C. tropicalis* isolates currently available in the MLST database, can be found at https://microreact.org/project/C.tropicalis. This public resource can be used interactively by end users to display and filter the results of this study on the basis of time, sample type, isolate, phylogeny, and geography.

## Discussion

In recent years, there has been an increase in the awareness that fungal infections represent an important problem not only for human health but also for the economy of the countries and their healthcare systems (Drgona et al., 2014; Denning, 2017^8^).

The global impact of *Candida* infections is enormous (Quindós, 2014; Enoch et al., 2017) although the epidemiology of the species involved in the invasive diseases is constantly changing as it depends on many factors including the geographical region considered, patient population, antifungal prophylaxis, diagnostic tests, and local hospital-related characteristics (Guinea, 2014; Quindós, 2014; Caggiano et al., 2015; Prigitano et al., 2016; Enoch et al., 2017; Epelbaum and Chasan, 2017). In this context, *C. tropicalis* has emerged as the predominant NAC species causing candidemia, especially in East Asian and Latin American countries (Yesudhason and Mohanram, 2015; Wang et al., 2016; da Matta et al., 2017; Motoa et al., 2017; Wu et al., 2017). In these countries, an extraordinary high incidence of *C. tropicalis* has also been recently reported from natural environments and animals (Chi et al., 2012; Brilhante et al., 2015; Cordeiro Rde et al., 2015; Zuza-Alves et al., 2016) by representing a potential threat to humans living in these geographical areas.

In Italy, candidemia is more common than in other European countries and has risen in incidence in the last few years (Bassetti et al., 2017). In the southern regions a dramatic increase of BSI caused by NAC species (Montagna et al., 2013; Delfino et al., 2014; Caggiano et al., 2015) has also been recently observed and *C. tropicalis* could represents an important cause of mortality in patients treated in our healthcare facilities (Montagna et al., 2013). In fact, during this study, *C. tropicalis* was the most common species recovered followed by *C. parapsilosis, C. albicans*, and *C. glabrata* (data not shown). Interestingly, in contrast to many other surveys (Kothavade et al., 2010; Nawrot et al., 2013; Quindós, 2014; Fernández-Ruiz et al., 2015;; Teo et al., 2017), in Italy, *C. tropicalis* BSIs seem to occur in patients without hematological malignancies (Montagna et al., 2013; Tortorano et al., 2013). This is also confirmed by our study in which all *C. tropicalis* isolates were recovered from individuals with neurological disorders. In addition we observed a specific correlation between the MLST genotypes recovered here and the geographic area and patient population considered. In fact all new Italian genotypes (DSTs 747-51 and 759) were not present in the *C. tropicalis* MLST database before and the unique deposited blood isolate with DST333 (the second most common genotype of this study) was previously reported from a Chinese patient hospitalized in a neurosurgery unit (Fan et al., 2014). However, a recent global analysis of the MLST genotypes confirmed a lack of association between different genotypes and antifungal resistance, specimen type, or geographical locations (Al-Obaid et al., 2017; Wu et al., 2017) even if the existence of an exclusive MLST clonal cluster from Italy suggests the occurrence of independent geographical evolution. Nevertheless, although the number of the isolates included in this study make up ∼20% (28/143) of the European *C. tropicalis* deposited in the MLST database, the amount of the examined Italian isolates was still too small to draw any solid conclusion and therefore further studies will be needed in order to corroborate our observations.

In this study, the most common genotype (DST747) was specifically associated with the ward C in which it was also found on a bed handle, a bedside table, and on the dominant hand of a social-health operator working in the unit (**Table 1**). The availability of genetic and epidemiological data (presence of catheters in our patients, time of hospitalization, and sharing of the same rooms) (Alanio et al., 2017) supports the evidence of an outbreak of *C. tropicalis* infection in our hospital and highlights a possible horizontal transmission of this specific Italian clone via contaminated hands of HCWs.

*Candida tropicalis* isolates from Italy showed a high degree of genetic homogeneity as only 8 DSTs were obtained from 28 examined isolates. This is in contrast with several other studies (Wu et al., 2012; Al-Obaid et al., 2017; Wu et al., 2017) and confirms the occurrence of healthcare-associated infections due to genetically closely related *C. tropicalis* isolates rather than a coincidental emergence of epidemiologically unrelated strains. In fact the nosocomial spread of *C. tropicalis* ceased just after the standard infection control measures were reinforced emphasizing the importance of active molecular surveillance programs in providing valuable information for the prevention and control of hospital infections.

## Conclusion

This study shows that the population structure of *C. tropicalis* is far from being fully elucidated as its complexity increases as different categories of patients and geographic areas are examined.

Based on clonal cluster analysis, Italian DSTs (333, 750, and 751) (**Figure 1**) appear to be more closely related to the Asian genotypes than those from Latin America or Europe, even if the number of isolates genotyped from these three continents is considerably variable. In fact, since the initial work of Tavanti et al. (2005), the *C. tropicalis* MLST database has not grown as rapidly as that for *C. albicans* which, at present, contains 3436 MLST profiles from over 4300 isolates (as of December 2017^9^). These two pathogenic *Candida* species are phylogenetically very closely related (Papon et al., 2013; Zuza-Alves et al., 2017) and share many common traits including a parasexual cycle (Seervai et al., 2013), white-opaque switching (Porman et al., 2011), and several other biological processes (Zuza-Alves et al., 2017) which could rapidly lead to the evolution and emergence of new strains as a result of the unstable and highly variable nature of the environmental conditions existing in the human hosts, hospitals, and in different geographic locations.

## Ethics Statement

This study was carried out in accordance with the recommendations of Ethics Committee of the IRCCS Centro Neurolesi Bonino Pulejo, Messina, Italy with written informed consent from all subjects. All subjects gave written informed consent in accordance with the Declaration of Helsinki. The protocol was approved by the ethical committee of the IRCCS Centro Neurolesi Bonino-Pulejo (study code: HCW_GR2011-02347606; protocol number: E12/17).

## Author Contributions

OR conceived the study. OR and FS designed the experiments. FS and GB collected the patient data, prepared the strains, and did phenotypic identification and antifungal susceptibility testing. FS, LG, FM, and MO performed the molecular experiments. LG and DG performed the bioinformatic analysis of the MLST data. OR, FS, LG, and DG interpreted MLST data and prepared figures and tables. OR wrote the main manuscript. FS and LG participated in discussions and provided suggestions. All authors reviewed and approved the final manuscript.

## Conflict of Interest Statement

The authors declare that the research was conducted in the absence of any commercial or financial relationships that could be construed as a potential conflict of interest.

## Footnotes

1 www.who.int/mediacentre/factsheets/en

2 https://pubmlst.org/calbicans

3 https://pubmlst.org/ctropicalis

4 www.eurofinsgenomics.eu

5 https://www.ncbi.nlm.nih.gov/BLAST

6 https://pubmlst.org/ctropicalis

7 https://microreact.org/project/C.tropicalis

8 www.gaffi.org

9 https://pubmlst.org/calbicans
